# Long-Term Survival of Adult Patients With Atrial Septal Defect With Regards to Defect Closure and Pulmonary Hypertension

**DOI:** 10.3389/fcvm.2022.867012

**Published:** 2022-04-28

**Authors:** Jana Rubáčková Popelová, Markéta Tomková, Jakub Tomek, Renata Živná

**Affiliations:** ^1^Department of Cardiac Surgery, Na Homolce Hospital, Prague, Czechia; ^2^Faculty Hospital Motol, Pediatric Heart Centre, Prague, Czechia; ^3^Department of Biochemistry and Molecular Medicine, School of Medicine, University of California, Davis, Davis, CA, United States; ^4^Department of Pharmacology, School of Medicine, University of California, Davis, Davis, CA, United States

**Keywords:** atrial septal defect, long-term survival, pulmonary hypertension, defect closure, congenital heart disease

## Abstract

**Background:**

Atrial septal defect (ASD) is the most common congenital heart disease (CHD) in adults and pulmonary hypertension (PH) is an established risk factor. A decision whether to perform ASD closure, especially in elderly patients with PH, is a complex dilemma. The aim of our study was to compare long-term survival in patients with closed and open ASD.

**Methods:**

A retrospective cohort study was performed on 427 patients with ASD (median age at diagnosis 38 years, IQR 18–56) out of which 186 patients (44%) manifested PH. ASD closure in patients with PH was only considered in patients without Eisenmenger syndrome with pulmonary vascular resistance < 5 WU. Median follow-up duration was 18 years (IQR 9–31 years). Kaplan-Meier and Cox proportional hazards survival analyses were performed to evaluate 12 potential predictors of survival.

**Results:**

Defect closure was associated with improved long-term survival in ASD patients both with (*P* < 0.001) and without PH (*P* = 0.01) and this association was present also in patients over 40 years. The 20-year survival since diagnosis was significantly higher in patients with PH and closed ASD compared to those with PH and open ASD (65% vs. 41%). ASD closure was a significant independent predictor of long-term survival (*P* = 0.003) after accounting for age at diagnosis, PH, NYHA class, Eisenmenger syndrome, and mitral regurgitation. Significant negative independent predictors of survival were older age at diagnosis (*P* < 0.001), Eisenmenger syndrome (*P* < 0.001), and PH (*P* = 0.03).

**Conclusion:**

ASD closure appears to be associated with improved long-term survival independently of age, PH, and other clinical variables.

## Introduction

Atrial septal defect (ASD) is the most common congenital heart disease (CHD) in adults and is often diagnosed late in adulthood. Identification of patients who would benefit from a closure of atrial septal defect (ASD) in adulthood remains a crucial, but complicated question (1). One particularly important group with unclear closure benefit comprises patients with pulmonary hypertension (PH). ASD closure appears safe in young patients without PH or even with PH meeting the criteria for defect closure (2–6). However, the presence of PH in older patients is associated with increased mortality following ASD closure compared to patients without PH (3, 4). It is not known whether the poor prognosis of the PH patients results from the ASD closure itself, or if their outcome would have been similar or even worse if the ASD closure was not carried out. As highlighted in the latest ESC guidelines, the impact of shunt closure on long-term outcome of patients with PH remains an area of uncertainty and requires further research (6).

We therefore sought to compare long-term survival of adults with and without ASD closure, with respect to PH, age, and other clinical variables.

## Methods

### Patients

Following institutional ethics committee approval, we performed a retrospective observational cohort analysis of isolated ASD patients in our database. The consecutive patient data were collected between 1995 and 2020. Mortality data were obtained from the national mortality register. The inclusion criteria were: adults with ASD type secundum, sinus venosus or coronary sinus defect diagnosed in adulthood or childhood, with accessible information concerning presence of PH and defect closure. The exclusion criteria were: ASD type primum, patent foramen ovale, missing data on ASD closure or on PH, or presence of another hemodynamically important congenital heart disease.

### Clinical Variables

For the purpose of this analysis, pulmonary hypertension (PH) was defined as mean pulmonary arterial pressure ≥ 25 mmHg, as determined by catheterization (if available) or echocardiography (7, 8). Patients suspected to have moderate or severe PH based on echocardiography have undergone right heart catheterization (RHC) with pulmonary vascular resistance (PVR) assessment. ASD size was measured by transesophageal echocardiography in 74% patients. The contraindications for ASD closure were: (a) Eisenmenger syndrome or non-indexed PVR > 5 Wood Units (WU) not responding to vasodilatation testing with epoprostenol or advanced therapy treatment (5, 6), (b) an increase in left atrial mean pressure during temporary balloon occlusion > 10 mmHg compared to baseline (9). In some patients, ASD was not closed in accordance with their wish.

### Statistical Analysis

Comparisons between cohorts (with and without PH) were performed with the Fisher’s exact test for binary variables and Mann-Whitney *U*-test for continuous variables, with Benjamini-Hochberg correction for multiple testing (false-discovery rate 0.05) applied on the reported *P*-values. Kaplan-Meier estimates and Cox proportional hazards model were used to analyze survival. All covariates were assessed that they fulfill the proportional hazards assumption, using the MATLAB fitcox function, which is based on the scaled Schoenfeld residuals, as derived by Grambsch and Therneau (10). The following variables were included in the univariable model: ASD closure, age at diagnosis, sex, New York Heart Association (NYHA) functional class, PH, moderate or severe mitral regurgitation (MR), Eisenmenger syndrome, ASD types (ASD secundum, sinus venosus, coronary sinus defect), ASD size, and advanced pulmonary vasodilator therapy. NYHA was used as a binary variable (NYHA > 2) to fulfill the proportional hazards assumption. The variables significant in univariable Cox proportional hazards model were then included in a multivariable model. Both the Kaplan-Meier and the multivariable Cox proportional hazards analyses were performed first in all patients (model A) and second in patients without Eisenmenger syndrome (model B). Values of *P* < 0.05 were considered statistically significant (^***^P < 0.001; ^**^P < 0.01; *P < 0.05). Statistical tests were two-sided. Data were analyzed using MATLAB (R2021b).

## Results

### Patient Characteristics

Data analysis was performed in 427 patients from our database meeting the inclusion criteria. Median age at diagnosis of the whole group was 38 (IQR 18–56) years and 74% of patients were female (Table 1). The age at diagnosis was higher in patients with PH compared to patients without PH: 50 (IQR 30–60) vs. 29 (IQR 13–46) years for closed defects (*P* < 0.001) and 53 (IQR 29–70) vs. 39 (IQR 28–57) for open defects (*P* = 0.3), (Table 1). Median follow-up was 18 years (IQR 9–31). Sinus venosus defect was present in 77 patients (18%) and coronary sinus defect in 8 patients (2%). Catheterization was performed in 60% of patients with PH and in 22% of patients without PH, altogether in 166 patients. ASD closure was performed in 367 patients (86%); out of which 58% by sternotomy, 14% video-assisted mini-thoracotomy, 10% robotic cardiac surgery, and 17% transcatheter.

**TABLE 1:** Patient characteristics.

Feature	No PH closed (*n* = 223)	PH closed (*n* = 144)	*P*-value closed no PH vs. PH	No PH open (*n* = 25)	PH open (*n* = 35)	*P*-value open no PH vs. PH	All (*n* = 427)
Age at diag. (years)	29 [13–46] (*n* = 217)	50 [30–60] (*n* = 150)	1 × 10^–8^ (***)	39 [28–57] (*n* = 24)	53 [29–70] (*n* = 36)	0.3	38 [18–56] (*n* = 427)
NYHA > 2	11.5% (25/217)	54.7% (82/150)	3 × 10^–18^ (***)	16.7% (4/24)	61.1% (22/36)	0.007 (**)	31.1% (133/427)
MR	12.0% (26/217)	28.9% (43/149)	1 × 10^–4^ (***)	12.5% (3/24)	31.4% (11/35)	0.2	19.5% (83/425)
Eisenmenger	*NA*	0.0% (0/150)	1	*NA*	19.4% (7/36)	0.1	1.6% (7/427)
ASD secundum	82.9% (180/217)	80.7% (121/150)	0.7	95.8% (23/24)	80.6% (29/36)	0.2	82.7% (353/427)
Sinus venosus	17.5% (38/217)	19.3% (29/150)	0.8	4.2% (1/24)	25.0% (9/36)	0.1	18.0% (77/427)
Coronary sinus	0.5% (1/217)	2.7% (4/150)	0.2	0.0% (0/24)	8.3% (3/36)	0.4	1.9% (8/427)
ASD size	16 [11–20] (*n* = 147)	20 [14–25] (*n* = 126)	0.001 (**)	6 [4–12] (*n* = 16)	19 [12–28] (*n* = 25)	9 × 10^–4^ (***)	18 [12–23] (*n* = 314)
Advanced therapy	0.0% (0/217)	4.7% (7/150)	0.003 (**)	0.0% (0/24)	8.3% (3/36)	0.4	2.3% (10/427)
Sex (male)	28.1% (61/217)	22.0% (33/150)	0.3	20.8% (5/24)	30.6% (11/36)	0.7	25.8% (110/427)
10-year survival	98.2% (166/169)	86.5% (109/126)	2 × 10^–4^ (***)	90.5% (19/21)	63.6% (21/33)	0.1	90.3% (315/349)
20-year survival	94.8% (110/116)	65.1% (56/86)	2 × 10^–7^ (***)	69.2% (9/13)	40.6% (13/32)	0.2	76.1% (188/247)
40-year survival	80.5% (33/41)	44.3% (27/61)	5 × 10^–4^ (***)	0.0% (0/5)	23.3% (7/30)	0.4	48.9% (67/137)
TVP	7.8% (17/217)	40.7% (61/150)	5 × 10^–13^ (***)	*NA*	*NA*	*NA*	*NA*
MVP + MVR	6.5% (14/217)	20.7% (31/150)	2 × 10^–4^ (***)	*NA*	*NA*	*NA*	*NA*
antiarrhythmic MAZE + CTI	4.6% (10/217)	24.0% (36/150)	2 × 10^–7^ (***)	*NA*	*NA*	*NA*	*NA*

Binary variables are given as percentage (positive/all cases). Continuous variables are given as median [interquartile range] (n), where n is the number of patients in the group with available data. ASD, atrial septal defect; MR, moderate or severe mitral regurgitation; NYHA, New York Heart Association class; PH, pulmonary hypertension; TVP, tricuspid valvuloplasty; MVP, mitral valvuloplasty; MVR, mitral valve replacement; CTI, ablation of cavo-tricuspid isthmus. (***P < 0.001; **P < 0.01; *P < 0.05).

### Pulmonary Hypertension

PH was present in 186 patients (44%), out of which 150 have undergone ASD closure (81%) and 36 have not (19%), (Table 1). The group of 36 patients with PH and open ASD comprised 12 patients who refused a recommended ASD closure (7 of them died), 7 patients with Eisenmenger syndrome (5 of them died), 4 patients with high PVR without Eisenmenger syndrome (3 died), 2 patients with small defects and PH (1 died), and 11 patients with various reasons for leaving ASD open (lung disease, left heart failure with high pulmonary capillary wedge pressure (PCW), age or increased PVR between 3 and 5 WU), 10 of them died.

### Concomitant Surgical Procedures

Concomitant surgical procedures were performed in moderate or severe valve regurgitations: tricuspid valve repair (TVP), mitral valve repair (MVP) or replacement (MVR) or documented supraventricular arrhythmias: MAZE procedure or cryo-ablation of cavo-tricuspid isthmus (CTI). The concomitant surgical procedures were significantly more frequent in the group of 150 closed defects with PH compared to the group of 217 closed defects without PH (TVP: 41% vs. 8%, MVP/MVR: 21% vs. 6%, MAZE/CTI: 24% vs. 5%, respectively, Table 1).

### Advanced Pulmonary Vasodilator Therapy

Advanced pulmonary vasodilator therapy was administered to 10 patients with ASD and PH in our study (5.4%): three patients with Eisenmenger syndrome and open ASD, six patients after ASD closure (all with persistent PH and PVR ≥ 2.9 WU), and one patient with PVR > 5 WU received the therapy both before and after the ASD closure. The remaining 4 patients with Eisenmenger syndrome died before the specific therapy was available.

### Pulmonary Hypertension and Atrial Septal Defect Closure as Predictors of Mortality

**FIGURE 1:**
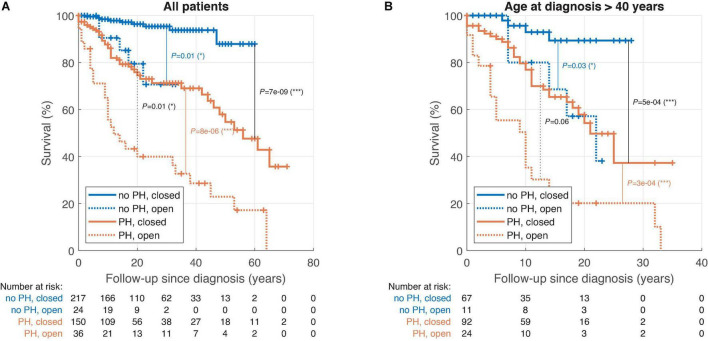
Kaplan-Meier survival analysis of atrial septal defect (ASD) patients stratified by pulmonary hypertension (PH) and ASD closure; all patients (model A). **(A)** Patients of all ages. **(B)** Patients diagnosed at the age above 40. ASD, atrial septal defect; MR, moderate or severe mitral regurgitation; NYHA, New York Heart Association class; PH, pulmonary hypertension.

As expected, PH was associated with higher all-cause mortality of ASD patients with closed defects (log-rank *P* < 0.001) as well as with open defects (log-rank *P* = 0.01 for model A and log-rank *P* = 0.006 for model B with Eisenmenger patients excluded) (Figure 1A and Supplementary Figure 1A). At the same time, defect closure was associated with improved survival not only in patients without PH (log-rank *P* = 0.01), but also in patients with PH and PVR < 5 WU (log-rank *P* < 0.001 for model A and log-rank *P* < 0.001 for model B) (Figure 1A and Supplementary Figure 1). Finally, this difference was present also in patients older than 40 years at diagnosis (Figure 1B and Supplementary Figure 1B).

The 20-year survival since diagnosis was higher in closed ASD than open ASD both in patients with PH (65% vs. 41%, *P* = 0.02) and without PH (95% vs. 69%, *P* = 0.01).

### Univariable Analysis of Mortality Prediction

**FIGURE 2:**
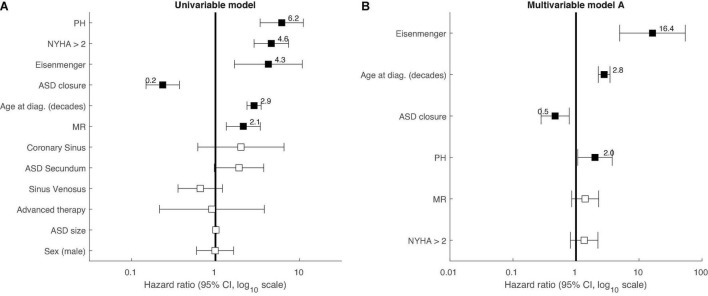
Forest plot of Cox-proportional hazards survival analysis in ASD patients. **(A)** univariable analysis. **(B)** Multivariable analysis (all patients, i.e., model A). The forest plot shows the hazard ratios and their 95% confidence intervals at a log_10_ scale. Significant variables are highlighted by a filled black square. ASD, atrial septal defect; MR, moderate or severe mitral regurgitation; NYHA, New York Heart Association class; PH, pulmonary hypertension.

Six variables significantly predicted mortality in a univariable model. While defect closure was negatively associated with mortality (hazard ratio 0.2, 95% confidence interval 0.1–0.4, *P* < 0.001), positive association with mortality was found for older age at diagnosis, PH, Eisenmenger syndrome, NYHA class, and MR (Table 2 and Figure 2A). The remaining variables were not significantly predictive of mortality: ASD secundum, sinus venosus defect, coronary sinus defect, advanced therapy, ASD size, and sex.

**TABLE 2:** Cox-proportional hazards models for mortality prediction.

	Univariable analysis (*n* = 427)		Multivariable model A (*n* = 425)		Multivariable model B (*n* = 418)	
Feature	Hazard ratio	*P*-value	Hazard ratio	*P*-value	Hazard ratio	*P*-value
Age at diag. (decades)	2.9 [2.4–3.5]	1 × 10^–27^ (***)	2.8 [2.3–3.5]	7 × 10^–22^ (***)	2.7 [2.2–3.4]	6 × 10^–20^ (***)
Eisenmenger	4.3 [1.7–10.8]	0.002 (**)	16.4 [4.9–54.3]	3 × 10^–06^ (***)		
ASD closure	0.2 [0.1–0.4]	2 × 10^–10^ (***)	0.5 [0.3–0.8]	0.003 (**)	0.5 [0.3–0.8]	0.003 (**)
PH	6.2 [3.4–11.1]	8 × 10^–10^ (***)	2.0 [1.1–3.8]	0.03 (*)	2.0 [1.0–3.7]	0.03 (*)
NYHA > 2	4.6 [2.9–7.4]	8 × 10^–11^ (***)	1.3 [0.8–2.2]	0.2	1.4 [0.8–2.3]	0.2
MR	2.1 [1.3–3.4]	0.001 (**)	1.4 [0.9–2.3]	0.2	1.4 [0.9–2.3]	0.2
ASD secundum	1.9 [1.0–3.8]	0.06				
Sinus venosus	0.7 [0.4–1.2]	0.2				
Coronary sinus	2.0 [0.6–6.6]	0.2				
Advanced therapy	0.9 [0.2–3.8]	0.9				
ASD size	1.0 [1.0–1.0]	0.4				
Sex (male)	1.0 [0.6–1.6]	1				

In the univariable analysis, all variables were assessed independently, and the significant variables were then included in the multivariable analysis (models A and B). In the multivariable model A, all 425 patients with non-missing values for the seven variables were included. In the multivariable model B, patients with Eisenmenger syndrome were excluded and all 418 patients with non-missing values for the remaining six variables were included. ASD, atrial septal defect; MR, moderate or severe mitral regurgitation; NYHA, New York Heart Association class; PH, pulmonary hypertension. (***P < 0.001; **P < 0.01; *P < 0.05).

### Multivariable Analysis of Mortality Prediction

The six variables significant in univariable analysis were subsequently included in a multivariable model A, in which four of them remained significantly predictive of mortality: ASD closure negatively (*P* = 0.003), while older age at diagnosis (*P* < 0.001), Eisenmenger syndrome (*P* < 0.001), and PH (*P* = 0.03) positively (Table 2 and Figure 2B). Finally, even when patients with Eisenmenger syndrome (as they are contraindicated for ASD closure) were completely excluded from this analysis (multivariable model B), the better long-term survival in patients with closed defects remained significant (*P* = 0.003) (Table 2).

## Discussion

The decision concerning ASD closure is particularly difficult in elderly patients with PH given the increased mortality risk in this group after ASD closure compared to young patients or patients without PH (3, 4, 11, 12).

The previous studies were missing a control group of patients without ASD closure, and thus could not distinguish whether the worsened survival is due to ASD closure itself, or PH and older age (3). Other previous studies have indicated that older patients with moderate or severe PH benefit from transcatheter closure of ASD with regards to improvement of symptoms and reduction of PH; however, these studies did not report a survival analysis (9, 13, 14).

A study of ASD and trisomy 21 showed a significantly higher mortality in uncorrected ASD including those with severe pulmonary vascular disease (PVD) compared to those with ASD closure. However, this study comprised only children and the group with PH before ASD closure was not analyzed (15).

Here, we conducted a retrospective study comparing long-term mortality in ASD patients with and without defect closure, with respect to PH, age, and other clinical variables. In line with previous studies (3, 4), we observed PH to be associated with higher all-cause mortality. The frequency of PH was high in our cohort (44%). This reflects the fact that our patients were older with late ASD diagnosis (they were followed-up since 1995). There could be also a selection bias resulting from the fact that high-risk patients with PH were sent to our tertiary referral center, while simple ASD patients were frequently treated at the local level.

A key finding of our study is that ASD closure (with adherence to the contraindication criteria) is associated with improved long-term survival in patients with or without PH and this holds also for patients over 40 years of age. ASD closure was a significant independent predictor of survival even after adjusting for age at diagnosis, PH, NYHA, MR, and Eisenmenger syndrome (which was present only in patients with open defects). In particular, ASD patients with PH had significantly better long-term survival after defect closure (65% after 20 years since diagnosis) compared to patients with PH and open ASD (41% after 20 years since diagnosis). Therefore, our data do not support the hypothesis that closure itself increases mortality risk for patients at high age or with PH, but rather that these patients are at high risk even without the closure. Our finding of the benefit of ASD closure is in line with results of a large nationwide study showing that patients with closed ASD have lower mortality compared to patients with open ASD (16).

It should be emphasized that the association of improved survival with ASD closure is relevant only for patients who fulfill the indication criteria for defect closure, with PVR ≤ 5 WU at baseline or after pulmonary vasodilator therapy (5, 6). Patients with PVR above 5 WU were shown to develop pulmonary arterial hypertension (PAH) late after defect closure, with poor prognosis (17). However, some case reports in the literature and also one of our patients suggest the possibility of “treat and repair” strategy with the use of advanced pulmonary vasodilator therapy in ASD patients with higher baseline PVR than 5 WU (18).

Patients with Eisenmenger syndrome are rare in ASD (1.6% in our study). Since Eisenmenger syndrome is a contradiction for defect closure, we designed our analysis with a special care to account for this potential confounding factor and performed all analyses with and without inclusion of patients with Eisenmenger syndrome. In our study, Eisenmenger syndrome was a strong predictor of mortality in univariable as well as in multivariable analysis (Table 2). However, ASD closure was a significant independent predictor of survival both in the analysis with Eisenmenger syndrome patients included (model A) and excluded (model B).

The presence of PH is sometimes erroneously considered to be a contraindication for defect closure on the basis of the study by Manes et al. (19). The authors observed the worst survival in patients with closed defects and PH, while the best survival was observed in Eisenmenger syndrome with open defects (most of them treated by advanced therapy). On the contrary, in our study, the highest long-term mortality was observed in open ASD with PH, including Eisenmenger syndrome. Eisenmenger syndrome, age at diagnosis, and PH were independent positive predictors of mortality in our study, while defect closure was a significant independent negative predictor of mortality. The discrepancy between the studies may be explained by the fact that the study of Manes et al. comprised predominantly post-tricuspid defects with unknown and potentially high preoperative PVR due to PAH and PVD. Therefore, the conclusions of Manes study are not applicable to ASD patients with PH and PVR ≤ 5 WU considered for closure. Moreover, the favorable long-term survival in patients with Eisenmenger syndrome in Manes et al. study could have been influenced by the use of advanced pulmonary vasodilator therapy. This therapy could only be used in a minority of our patients. While we observed general stabilization of clinical state in patients treated with advanced therapy, the numbers were too low to evaluate a potential significant benefit in the survival analysis.

The results of our study support the strategy of active screening of the ASD in adults with timely closure of hemodynamically significant defects early after diagnosis. ASD closure should be performed even in asymptomatic patients and on the other side even in older patients with PH if they comply with the indication criteria for ASD closure specified in the guidelines (5, 6). Transcatheter closure is preferred because of its lower invasiveness; however, if a patient with ASD and PH has concomitant severe tricuspid or MR or large defect without appropriate rims, we believe there is still place for surgery as recommended by the guidelines (5, 20).

High-risk patients should always be assessed for risks and benefits of ASD closure in a specialized expert center (6). ASD closure may prevent further deterioration of PH and improve survival, however in patients with severe PH and advanced PVD, the shunt closure may be detrimental. Therefore, the catheterization with PVR assessment is crucial (5, 6, 21). The current recommended cut-off value of PVR for ASD closure is 5 WU (6). In high-risk patients with PAH, a small fenestration may be useful to prevent right heart failure after ASD closure. In postcapillary and combined PH, we aimed to remove the cause of postcapillary PH (mitral regurgitation, coronary artery disease, left heart failure, etc.) before or during the ASD closure and to leave a small fenestration to prevent left heart failure.

Mortality and morbidity (stroke or heart failure) are significantly increased by atrial arrhythmias (22). The risk of atrial arrhythmias and stroke is increased in patients with ASD (23, 24) and the prevalence of atrial arrhythmias in ASD increases with age (22). In our study, we performed the antiarrhytmic surgery (MAZE and/or CTI) in the case of documented atrial arrhythmias. This procedure was significantly more frequent in patients with PH compared to defects without PH (24% vs. 5%, *p* < 0.001). Close long-term follow-up of patients with open as well as closed ASD is important. It should include clinical examination with assessment of symptoms, especially arrhythmias, repeated echocardiogram, and ECG monitoring. Some of the small defects may increase their hemodynamic relevance with increasing age and such defects should be closed in time.

Our study confirmed that ASD patients with PH had higher mortality and suffered more often from mitral and tricuspid regurgitation and arrhythmias, compared to patients without PH. This is in line with the literature; patients with PH had more comorbidities, were older and had a significantly higher risk of developing major cardiac and cerebrovascular adverse events after transcatheter ASD closure (25).

In conclusion, our study on 427 ASD patients identified ASD closure as a positive independent predictor of survival. In contrast, older age at diagnosis, Eisenmenger syndrome, and PH were independent negative predictors of survival. Patients with PH and closed ASD had significantly lower long-term mortality compared to patients with PH and open defects. ASD closure thus appears to be beneficial even in older patients with PH and PVR < 5 WU, who are not contraindicated for closure for other reasons.

## Limitations of the Study

(A) Due to the retrospective nature of our study, the groups with and without ASD closure could not be matched. Patients with open ASD and PH formed a heterogenous group comprising patients contraindicated for ASD closure (Eisenmenger syndrome, small defects with severe PH), and patients who refused ASD closure despite PVR < 5 WU. However, the mortality was high in all subgroups of open ASD and PH patients. (B) While the long-term follow-up (and data collection since 1995) is of a general advantage of our study, it brings two inherent limitations. First, catheterization was not performed in all patients, but preferentially in those with suspicion of moderate or severe PH based on echocardiography. Second, not all patients with high PVR had the possibility of advanced pulmonary vasodilator therapy treatment. However, as soon as the advanced therapy was accessible, it was administered to all patients with Eisenmenger syndrome or high PVR who were still alive. The impact of advanced vasodilator therapy on the long-term survival of ASD patients with high PVR and open or closed defects therefore remains to be assessed in future research.

## Data Availability Statement

The raw data supporting the conclusions of this article will be made available by the authors, without undue reservation.

## Ethics Statement

The studies involving human participants were reviewed and approved by Ethics Committee of Na Homolce Hospital, project 5.6.2019/25 and 26. Written informed consent for participation was not required for this study in accordance with the national legislation and the institutional requirements.

## Author Contributions

JR conceived the study, collected the patient data, and was the guarantor of the study. RŽ contributed to data collection. MT and JT carried out data analysis and visualization. MT and JR wrote the initial draft, with all authors subsequently carrying out critical revisions.

## Conflict of Interest

The authors declare that the research was conducted in the absence of any commercial or financial relationships that could be construed as a potential conflict of interest.

## Publisher’s Note

All claims expressed in this article are solely those of the authors and do not necessarily represent those of their affiliated organizations, or those of the publisher, the editors and the reviewers. Any product that may be evaluated in this article, or claim that may be made by its manufacturer, is not guaranteed or endorsed by the publisher.
